# An Evaluation of SmokeFree for Kansas Kids: An Intervention to Promote Tobacco Cessation in Pediatric Clinics

**Published:** 2017-02-15

**Authors:** Thanuja Neerukonda, Taneisha S. Scheuermann, Stephen J. Lauer, Melissa Hudelson, Edward F. Ellerbeck

**Affiliations:** 1 University of Missouri-Kansas City School of Medicine, Kansas City, MO; 2 Department of Preventive Medicine and Public Health, University of Kansas Medical Center, Kansas City, KS; 3 Department of Pediatrics, University of Kansas Medical Center, Kansas City, KS; 4 Kansas Chapter, American Academy of Pediatrics, Lenexa, KS

**Keywords:** smoking cessation, pediatrics, tobacco smoke pollution

## Abstract

**Introduction:**

Smokefree for Kansas Kids is a program designed to train pediatric clinic staff to assess for tobacco exposure and provide brief smoking cessation interventions to caregivers and patients. The purpose of this study was to evaluate the impact of this program and improve future tobacco intervention efforts in pediatric clinics.

**Method:**

Eighty-six pediatric physicians and staff attended at least one of three training sessions. A random sample of pediatric medical records was selected pre-intervention (n = 49) and post-intervention (n = 150). Electronic medical records were reviewed to assess for documentation of tobacco use intervention implemented in the clinic.

**Results:**

Of the 199 pediatric clinic visits reviewed, 197 met the study criteria. All but one visit documented an assessment of tobacco exposure. Among children exposed to tobacco (n = 42), providers were more likely to discuss tobacco use with caregivers post-intervention (35.7%) compared to pre-intervention (7.1%; p < 0.05). One in five caregivers in the post-intervention group were advised to quit (21.4%) compared to the pre-intervention group (7.1%). In the post-intervention group, 14.3% were referred to the state quitline compared to no referrals in the pre-intervention group. The difference in rates for providing advice and referral between pre-intervention and post-intervention were not statistically significant.

**Conclusions:**

Implementation of the Smoke Free for Kansas Kids intervention was associated with modest improvements in clinic tobacco intervention efforts, but many patients still failed to receive optimal assessments or interventions. Additional efforts may be needed to enhance this program.

## Introduction

Tobacco use is the leading and most preventable cause of death and illness throughout the United States.^1^ Smoking harms almost every single organ in the body. Despite the rates of decline in smoking throughout the years, nonsmokers remain exposed to tobacco smoke in homes, vehicles, and public places.^2^, ^3^ Many of these nonsmokers are children. One out of five children live with someone who smokes in the household.^4^ More than 50% of children between three and eleven years of age have detectable levels of tobacco-specific biomarkers due to secondhand smoke exposure.^4^ Secondhand smoke exposure is associated with an increased prevalence of many negative health outcomes including severe asthma, upper respiratory symptoms and infections, ear infections, and increased risks for sudden infant death syndrome (SIDS).^3^ One of the greatest risk factors for smoking initiation in youth is parental smoking.^5^

Pediatric clinic visits provide an opportunity to reduce secondhand smoke exposure for children. In fact, parents expect pediatricians to address smoking and the majority of them want information on smoking cessation.^6^,^7^,^8^ However, secondhand smoke exposure is not assessed routinely in pediatric clinics.^9^,^10^ Physicians, particularly pediatricians, often fail to record tobacco use information in the electronic medical record (EMR).^11^ Use of EMRs can strengthen health care providers’ abilities to identify and intervene on tobacco use,^11^ and integration of tobacco exposure screening in health record documentation can improve rates significantly.^12^,^13^ Therefore, training clinic staff to intervene with parents and documenting tobacco exposure is important in reducing children’s secondhand smoke exposure.

To reduce the adverse effects of smoking on children, a number of efforts have been undertaken to incorporate smoking cessation interventions into pediatric settings.^6^,^14^ Kids Safe and Smokefree (KiSS),^15^ Stop Tobacco Outreach Program,^16^ and CEASE^17^ are examples of pediatrician-parent interventions for tobacco cessation. These interventions involved advising parents to quit, referring them to quitlines, and following up after intervention. Importantly, KiSS and CEASE included office system changes to support delivery of tobacco treatment. Previous research found that, after receipt of training on tobacco treatment, providers’ delivery of tobacco treatment decreased over time without “booster” trainings.^18^ Office systems, including electronic medical record prompts, offer promise to support the delivery of tobacco treatment.^12^,^19^

KiSS was a multilevel intervention evaluated in a randomized trial.^8^ The clinic-level intervention components included modifying the electronic health record screens to provide guideline-based tobacco intervention prompts to remind providers to ask about child secondhand smoke exposure, advise about the risks of secondhand smoke exposure, and refer smokers to cessation programs. This intervention was combined with telephone-based behavioral counseling. If shown to be effective, KiSS will provide a comprehensive model for addressing the issue of secondhand exposure to tobacco smoke in the pediatric population.^15^

The Stop Tobacco Outreach Program was a program to intervene with smoking parents of children admitted to the hospital for a respiratory illness.^16^ This intervention integrated motivational interviewing, nicotine replacement therapy, telephone counseling, and referral. At two-month follow-up, fewer parents reported smoking in homes or cars and there was a significant increase in attitudes acknowledging the harms of smoke exposure.

The CEASE intervention included routine screening for parental tobacco use, motivational messaging, nicotine patch/gum recommendations, and enrollment in free state quitlines.^10^ In a recent study, this intervention was implemented and evaluated in 10 pediatric clinics and compared to 10 control pediatric clinics. Pediatric clinics that implemented CEASE had a higher rate of providing tobacco treatment counseling (42.5%) compared to control clinics (3.5%). The effect of the CEASE trainings and system-changes on tobacco treatment delivery were still evident one year post-intervention.^12^ These results showed that programs such as CEASE can be implemented successfully in child health care settings.^12^, ^17^

The Kansas Chapter of the American Academy of Pediatrics (KAAP) with the University of Kansas Medical Center (KUMC) Department of Pediatrics developed an intervention program based on CEASE^17^ called Smokefree for Kansas Kids that was funded through the Kansas Health Foundation. This intervention program involved training clinic staff to conduct tobacco use assessments consistently, advise parents to quit, and provide referrals to the state tobacco quitline. It also modified the EMR to provide tools to enhance the evaluation of secondhand smoke exposure, the sources of this exposure, and prompt tobacco treatment. The aim of this study was to assess the effectiveness of the Smokefree for Kansas Kids program after the first year of implementation by evaluating the pre-intervention to post-intervention EMR documentation changes.

## Methods

### Intervention

KAAP and KUMC worked together to develop the first year of the Smokefree for Kansas Kids intervention program. This program included three trainings in the first year. Training involved the introduction of the intervention program, explanation of the three steps to clinic-based tobacco intervention, training on motivational interviewing, information on KanQuit (the free tobacco quitline for the state of Kansas), and information on cessation medications.

Smokefree for Kansas Kids adopted the brief tobacco treatment method using Ask, Advise, Refer tobacco treatment components^20^ based on the CEASE program that successfully has been implemented elsewhere.^17^ The Ask component involved asking families at every health encounter about tobacco use and rules about smoking within the home and car (e.g., “Does your child live with anyone who uses tobacco?”). The Advise component involved giving families strong, clear, personalized advice. The trainings emphasized discussing the impact of smoking on finances as well as the health of the child and other members of the family. For example, “Quitting smoking is one of the most important things for your own health and your child’s health. I can help you quit.” The Refer component involved referring the family members who use tobacco to KanQuit and informing the family that each referral to KanQuit will be rewarded with a $40 gift card. Fax referrals were made through KanQuit and smokers were contacted once the referral form was received. For most callers, the program offered proactive counseling sessions and follow-up assistance.^21^ The training also provided participants with detailed explanations of multiple smoking cessation medications, such as nicotine replacement, non-nicotine treatments, and combination medications. The trainings emphasized recording tobacco use in the child’s electronic medical record and documenting smoke exposure on the problem list.

In addition to training in the Ask, Advise, and Refer components of tobacco treatment, clinic staff received brief training on motivational interviewing. Because counseling is more effective when it is delivered in a non-judgmental manner,^22^ this training introduced brief counseling skills, such as asking open-ended questions and using feedback to confirm the meaning of what the caregivers are saying. Training also addressed expressing empathy and enhancing caregiver confidence to quit smoking. Training included the Elicit-Provide-Elicit process to increase interest in tobacco cessation information: Elicit: ask permission before providing information (e.g., “Would you like to learn more about…?”), Provide: provide feedback in a neutral manner (e.g., “What happens to some people is…”), Elicit: obtain the patient’s interpretation and follow-up (e.g., “What do you make of this?”).^22^

Three trainings were offered between September 2014 and April 2015. Each training session lasted for one hour. Eighty-six attendees attended at least one session of training.

### Participants

Charts were selected for review based on dates of visits. Forty-nine patients were selected randomly for EMR analysis prior to the intervention (February – May 2014) and 150 were selected randomly for EMR analysis after the intervention (September 2014 – June 2015). The 150 post-intervention visits were selected in groups of 50; clinic visit dates were within the three months following each of the three clinic trainings. The evaluation period followed the initial training session because this training covered the brief tobacco treatment intervention and was designed to be immediately implemented by providers. The two additional trainings offered later in the year were designed to reinforce and expand on the skills learned. Therefore, visits following these trainings were sampled to ensure that the evaluation included any effects of these subsequent sessions. Inclusion criteria included pediatric patients aged from newborn to 17, seen in the KUMC pediatric clinic. Medical records included well child visits and office visits; visits where no progress notes were recorded in the text fields (e.g., for injections) were excluded.

For each chart reviewed, the following descriptors of the visit were recorded: patient’s age, date of outpatient visit, visit type, and provider specialty. The charts were reviewed for tobacco exposure and evaluation of provided tobacco treatment. Whether tobacco use was mentioned in progress notes, whether tobacco exposure was included in the EMR problem list, whether smoking was addressed in written patient instructions, whether smokers were advised to quit, and whether fax referral forms to the quitline were present in the EMR were recorded. The study protocol was approved by the KUMC Institutional Review Board.

### Measures

Data collected from each reviewed chart were entered into REDCap.^23^ Tobacco exposure status was obtained from the social history in the EMR for each visit. The primary outcome was the change in the use of each component of the 3-step (Ask, Advise, and Refer) tobacco intervention for visits with tobacco exposed children. Information was collected on whether tobacco use was discussed during the office visit. “Ask” was defined as whether tobacco exposure was assessed. “Discussed” was defined as whether tobacco exposure was mentioned in the progress notes or included on the problem list of the EMR. “Advise” was defined as whether the provider documented advising the smoker to quit in the EMR. Therefore, the number of medical records that identified any advice to quit was recorded pre-intervention and post-intervention. “Refer” was defined as whether the clinician documented offering any method of follow-up counseling, referral to the state quitline, or prescription medications for smoking cessation.

### Analyses

Descriptive statistics such as frequencies and percentages were calculated to determine tobacco treatment practices before and after the intervention. Comparisons were made between pre-intervention and post-intervention data regarding tobacco use assessment, discussion of tobacco exposure, advice to quit, and referral. Mid-P exact probability tests with one tail p-values were calculated for each component of Smoke Free for Kansas Kids, using openepi.com to determine whether there were statistically significant increases in tobacco treatment from pre- to post-intervention.

## Results

**Figure 1:**
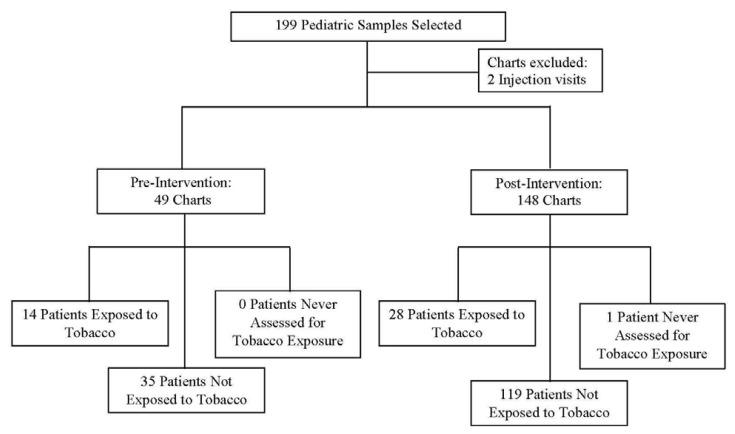
Sample for chart review and tobacco exposure status.

Figure 1 illustrates the pediatric samples for the project. Of the 199 pediatric visits identified, two visits were excluded, which were for injections. Of the remaining 197, 49 samples were pre-intervention charts and 148 were post-intervention charts. From the 49 pre-intervention charts, all patients were assessed for tobacco exposure. Fourteen of the 49 patients were exposed to tobacco and 35 patients were not exposed to tobacco. From the 148 post-intervention charts, one patient was not assessed for tobacco exposure, 28 patients were exposed to tobacco use, and 119 patients were not exposed to tobacco.

Table 1 provides the number and percentage of visits with documentation of the three components of the intervention: Ask, Advise, and Refer for the pre-intervention and post-intervention groups. The post-intervention group had higher rates of tobacco-related discussions (35.7%) in comparison to the pre-intervention group who had a rate of 7.1%. In addition, the post intervention group also received tobacco use counseling at a higher rate; 21.4% of adult caregivers who used tobacco were advised to quit and 14.3% were referred to the quitline in comparison to pre-intervention (7.1% for advice to quit and 0% for referral to quitline).

## Discussion

This study demonstrated the feasibility of adopting intervention programs to reduce secondhand smoke exposure through implementation of an intervention program such as Smokefree for Kansas Kids. Before implementation of Smokefree for Kansas Kids, the rate of documented tobacco use and assessment and tobacco treatment was very low. After implementation, tobacco use assessment and documentation in the progress notes and problem list by health care providers increased. While the rates for providing advice and referral appear higher post-intervention, the differences were not statistically significant. However, given the small sample size of tobacco-exposed patients included in this study, our findings provide preliminary evidence for the feasilbity of implementing clinic-based tobacco treatment intervention in Kansas pediatric clincs.

While our findings indicated that implemention of smoking cessation programs may be feasible, they also highlighted the need for further improvement in routinely assessing and treating tobacco use exposure within pediatric health care settings. Even after implementation of Smokefree for Kansas Kids, less than 50% of the pediatric visits with children exposed to tobacco smoke included documented tobacco assessment and treatment by the health care provider. Even though the rates increased between pre- and post-intervention, the numbers are low, especially with regard to tobacco treatment. In the second phase of Smokefree for Kansas Kids, we plan to provide additional trainings as well as implement EMR changes. Altering pediatric EMRs to include specific tobacco assessment and treatment questions could improve the rates of smoking assessment and treatment further.

This study was limited by reliance on restrospective chart reviews to evaluate tobacco assessment and treatment. Some aspects of important data may not have been available for research purposes. We assumed that tobacco exposure was assessed if the EMR showed an updated tobacco exposure status as of that visit. However, changes in EMR documentation does not necessarily indicate real changes in care occurred; it is possible that the trainings stimulated improved documentation, but rates of assessment, advice, and referral did not change from pre-intervention to post-intervention. On the other hand, discussions also may have occurred that were not documented in the EMR.

## Conclusions

Our results showed that a pediatric clinic intervention including the Ask, Advise, and Refer tobacco treatment model, motivational interviewing, and training regarding quitlines and smoking cessation mediciations can be implemented in pediatric clinic settings. However, there is room for improvement based on the fact that no intervention was recorded for greater than half of patients exposed to secondhand smoke. These results were used to inform additional trainings and specific ideas for EMR changes for the second year of the Smoke Free for Kansas Kids project. Future studies and evaluations need to be conducted to establish how components of the intervention could be implemented and sustained successfully among physicians, nurse practitioners, nurses, and medical assistants in pediatric clinics to promote reduction of secondhand smoke exposure and smoking cessation among adult caregivers.

## Figures and Tables

**Table 1:** Tobacco treatment pre- and post-intervention for patients exposed to tobacco smoke.

Action	Pre-intervention n = 14	Post-intervention n = 28	p-Value
*Discussed Tobacco Use*			
Tobacco Exposure Addressed in Progress Notes	1 (7.14%)	10 (35.71%)	0.027
Tobacco Exposure Addressed in Problem List	1 (7.14%)	8 (28.57%)	0.064
*“Advise” Component of SFKK*			
Advised Smokers to Quit	1 (7.14%)	6 (21.43%)	0.142
*“Refer” Component of SFKK*			
Discussion of Quitline, Follow-up Counseling, or Smoking Cessation Medications.	(0) 0%	4 (14.28%)	0.0915
